# Overexpression of *GPC6* and *TMEM132D* in Early Stage Ovarian Cancer Correlates with CD8+ T-Lymphocyte Infiltration and Increased Patient Survival

**DOI:** 10.1155/2015/712438

**Published:** 2015-09-13

**Authors:** Athanasios Karapetsas, Antonis Giannakakis, Denarda Dangaj, Evripidis Lanitis, Spyridon Kynigopoulos, Maria Lambropoulou, Janos L. Tanyi, Alex Galanis, Stylianos Kakolyris, Gregorios Trypsianis, George Coukos, Raphael Sandaltzopoulos

**Affiliations:** ^1^Laboratory of Gene Expression, Molecular Diagnosis and Modern Therapeutics, Department of Molecular Biology and Genetics, Faculty of Health Sciences, Democritus University of Thrace, 68100 Alexandroupolis, Greece; ^2^Division of Genome and Gene Expression Data Analysis, Bioinformatics Institute, Agency for Science, Technology and Research (A∗STAR), Singapore 138671; ^3^Department of Oncology, University Hospital of Lausanne (CHUV), Ludwig Cancer Research Center, University of Lausanne, 1011 Lausanne, Switzerland; ^4^Laboratory of Histology and Embryology, School of Medicine, Faculty of Health Sciences, Democritus University of Thrace, 68100 Alexandroupolis, Greece; ^5^Division of Gynecologic Oncology, University of Pennsylvania, Philadelphia, PA 19014, USA; ^6^Department of Clinical Oncology, School of Medicine, Faculty of Health Sciences, Democritus University of Thrace, 68100 Alexandroupolis, Greece; ^7^Medical Statistics Lab, School of Medicine, Faculty of Health Sciences, Democritus University of Thrace, 68100 Alexandroupolis, Greece

## Abstract

Infiltration of cytotoxic T-lymphocytes in ovarian cancer is a favorable prognostic factor. Employing a differential expression approach, we have recently identified a number of genes associated with CD8+ T-cell infiltration in early stage ovarian tumors. In the present study, we validated by qPCR the expression of two genes encoding the transmembrane proteins GPC6 and TMEM132D in a cohort of early stage ovarian cancer patients. The expression of both genes correlated positively with the mRNA levels of *CD8A*, a marker of T-lymphocyte infiltration [Pearson coefficient: 0.427 (*p* = 0.0067) and 0.861 (*p* < 0.0001), resp.]. *GPC6* and *TMEM132D* expression was also documented in a variety of ovarian cancer cell lines. Importantly, Kaplan-Meier survival analysis revealed that high mRNA levels of GPC6 and/or TMEM132D correlated significantly with increased overall survival of early stage ovarian cancer patients (*p* = 0.032). Thus, *GPC6* and *TMEM132D* may serve as predictors of CD8+ T-lymphocyte infiltration and as favorable prognostic markers in early stage ovarian cancer with important consequences for diagnosis, prognosis, and tumor immunobattling.

## 1. Introduction

Despite the slight decrease in the incidence and death rates of ovarian cancer over the recent years, it remains the major cause of death due to gynecological malignancies. About 22,000 new cases and 14,000 deaths are expected in the United States alone in 2014 [1]. Because of the lack of obvious and specific symptoms at the onset of the disease, the majority of the cases are diagnosed at a late stage. The five-year overall survival rate of ovarian cancer patients is approximately 44%. On the contrary, patients with early stage ovarian cancer exhibit significantly higher survival rates [2]. The age at the time of diagnosis, the stage of the disease, the histological subtype, and tumor grade are common prognostic factors used to predict clinical outcome [3]. Similarly, the expression levels of several genes have been found to correlate with patients' survival [4–7]. For instance, patients with low/intermediate levels of* BRCA1* mRNA exhibit higher overall survival following treatment with platinum-based chemotherapy compared to patients with high levels of* BRCA1* mRNA [8]. Thus, characterization of molecular markers with prognostic value is of great importance in order to stratify high-risk ovarian cancer patients and implement the most appropriate therapeutic scheme.

Similar to several other types of solid cancers, ovarian tumors are immunogenic with various immune cell populations infiltrating the tumor sites. Zhang et al. demonstrated that intratumoral infiltration of CD3+ T-lymphocytes correlates significantly with high progression-free and overall survival of ovarian cancer patients [9]. Since then, a number of studies have highlighted the prognostic significance of T-cell infiltration in ovarian cancer [10–13]. For instance, it has been well documented that infiltration with high numbers of CD8+ T-lymphocytes associates positively with survival benefit and favorable clinical outcome [14, 15]. So far, gene expression profiling by microarrays has been employed by three independent research groups in order to elucidate the genes and the underlying molecular mechanisms that govern T-cell infiltration in ovarian cancer [16–18]. All of these studies focused on advanced stage ovarian cancer and each identified a number of differentially expressed genes that were associated with CD8+ T-lymphocyte infiltration and immune responses and even with survival [16–18].

Recently, utilizing a fluorescent version of the ADDER (Amplification of Double-Stranded cDNA End Restriction Fragments) Differential Display methodology, we identified, for the first time, genes overexpressed in early stage ovarian cancer which are associated with CD8+ T-lymphocyte infiltration [19]. For instance, the mRNA levels of one of the identified genes,* SMARCE1*, correlated significantly with the expression of* CD8A*, a marker of T-cell infiltration. Importantly, forced overexpression of* SMARCE1* in ovarian cancer cells induced the expression and secretion of certain chemokines and consequently triggered the chemotaxis of CD8+ T-lymphocytes* in vitro* [19].

In the present study, we evaluated the expression of two other overexpressed genes,* GPC6* and* TMEM132D*, and investigated whether they could represent novel prognostic markers of survival in early stage ovarian cancer. We selected to study these two genes as they are both surface antigens. TMEM132D is a transmembrane protein, while GPC6 is a GPI-anchored protein on the outer surface of the cell which may be cleaved off and released in the extracellular space. Through the heparan sulfate glycosaminoglycan chains GPC6 can interact with other molecules and receptors of the membrane. Moreover, glypicans may regulate Hedgehog, Wnt, BMP, and FGF signaling pathways. On the contrary, the exact biological role of TMEM132D still remains quite elusive. The localization of these proteins suggests that they may be involved in intercellular signalling and cell-cell recognition, aspects critical for the mechanisms of cell attraction and recruitment. We validated with qPCR the expression of* GPC6* and* TMEM132D* in a cohort of stage I-II ovarian cancer patients. The expression of both genes correlated positively with the* CD8A* marker and thus with T-cell infiltration. Furthermore, the expression of both genes was monitored in a variety of ovarian cancer cell lines. Ultimately, we performed a retrospective survival analysis of the early stage ovarian cancer patients and correlated the mRNA levels of* GPC6* and* TMEM132D* with the overall survival. Patients with high mRNA levels of* GPC6* and/or* TMEM132D* exhibited survival benefit compared to patients with low mRNA levels of both genes.

## 2. Materials and Methods

### 2.1. Patients and Specimens

Ovarian cancer tumor specimens were obtained from patients undergoing primary debulking surgery by surgeons within the Gynecologic Oncology Division at the University of Pennsylvania. The stage of the disease was determined by the gynecologic oncologists. The histology and grade of the tumor samples were established by the surgical pathologist. Specimens were immediately snap-frozen and stored at −80°C. The tissue collection was approved by the IRB committee of the University of Pennsylvania. The analysis of the samples took place at the Department of Molecular Biology and Genetics, abiding to the guidelines of the Ethics Commission of the Democritus University of Thrace.

### 2.2. Cell Lines and Culture

Human epithelial ovarian cancer cell lines SKOV3, OVCAR3, OVCAR5, A1847, A2780, and C30 were acquired from ATCC and cultured in RPMI 1640 with stable glutamine, supplemented with 10% FBS, 100 U/mL penicillin, and 100 *μ*g/mL streptomycin (all from Biosera). Mouse ovarian cancer cell line ID8 was originally donated by Drs. Kathy Robby and Paul Terranova and cultured in DMEM high glucose with stable glutamine and sodium pyruvate (Biosera), supplemented with 10% FBS, 100 U/mL penicillin, and 100 *μ*g/mL streptomycin. Cell lines were cultured at 37°C, 5% CO_2_, in a humidified atmosphere and passaged for fewer than six months since receipt and stock thawing.

### 2.3. RNA Isolation and Reverse Transcription

Total RNA was isolated from tissue or from 1 × 10^6^ cells with TRIzol reagent (Life Technologies). The quality, integrity, and quantity of the isolated RNA were assessed spectrophotometrically and by gel electrophoresis. Following treatment with RNase-free DNase, total RNA was reverse-transcribed using Superscript First-Strand Synthesis Kit for RT-PCR (Life Technologies) according to manufacturer's instructions.

### 2.4. Quantitative PCR

qPCR was performed on a StepOne real-time PCR system (Applied Biosystems) using the KAPA SYBR Fast qPCR kit (KAPA Biosystems) under the following conditions: 95°C/3 min and then 40 cycles at 95°C/15 seconds and at 59°C/1 min. The housekeeping gene* b-actin* was employed as internal control for normalization. The primers for* CD8A*,* GPC6*,* TMEM132D*, and* b-actin* were as follows:

 
*CD8A* forward 5′-CCCTGAGCAACTCCATCATGT-3′ and
 reverse 5′-GGCTTCGCTGGCAGGAA-3′,
 
*GPC6* forward 5′-GGGCACAGCAAAGCCAGATA-3′ and
 reverse 5′-TGGTTGGTGAGCCCATCAT-3′,
 
*TMEM132D* forward 5′-CACTGGTCGCCGGTACTCAT-3′ and
 reverse 5′-GACCTTCCGTCACTTTGGAAAA-3′,
 
*b-actin* forward 5′-GCGCGGCTACAGCTTCA-3′ and
 reverse 5′-CTTAATGTCACGCACGATTTCC-3′.

For relative quantification, the formula RQ = 2^−(ΔΔCt)^ was used. Prior to using the ΔΔCt method for relative quantification of the transcripts, validation experiments were performed by applying the relative standard curve method in order to demonstrate that the PCR efficiencies of the targets* CD8A*,* GPC6*, and* TMEM132D* and of the housekeeping gene* b-actin* were approximately equal. In general, each reaction was run in triplicate and each PCR experiment included two nontemplate controls.

### 2.5. ADDER Fluorescent Differential Display

ADDER was adapted from [20] for fluorescent detection as previously described [19].

### 2.6. Immunohistochemistry

Paraffin embedded tissue samples from ovarian tumors were available from patients who underwent surgery. Four-micron sections (4 *μ*m) of representative blocks from each case were deparaffinized, rehydrated, and treated with 0.3% H_2_O_2_ for 5 min in methanol to prevent endogenous peroxidase activity and were immunostained by the peroxidase method (Envision System, DAKO, Carpinteria, California, USA) according to the manufacturer's recommendations. After antigen retrieval and endogenous peroxidase blockade, the sections were incubated overnight at 4°C with polyclonal antibodies against Glypican-6 (Acris, Germany) and TMEM132D (Novus Bio, USA) in 1 : 100 and 1 : 80 dilutions, respectively. Then, the sections were incubated with secondary antibody at room temperature for 60 min. Finally, bound antibody complexes were stained for 10 min with 0.05% diaminobenzidine. Sections were then briefly counterstained with Mayer's haematoxylin, mounted, and examined under a Nikon Eclipse 50i microscope. Control slides were incubated for the same period with nonimmunized rabbit serum (negative control). A positive control was always run in the assay. The staining results were evaluated by a pathologist based on the percentage of staining in tumor cells.

### 2.7. Statistical Analysis

Graphs and statistical analysis of the data were performed with GraphPad Prism 5 and SPSS version 19.0. Correlation of* GPC6* and* TMEM132* mRNA levels with* CD8A* expression levels was examined by Pearson coefficient correlation. The chi-square test was used to assess the association of the expression levels of* GPC6* and* TMEM132D* with patients' clinicopathologic characteristics (stage, grade, and histotype). To study whether mRNA levels of* GPC6* and* TMEM132D* were predictive for overall survival, survival rates were calculated with the Kaplan-Meier method and the statistical difference between survival curves was determined with the log-rank test. Overall survival was defined as the time interval from diagnosis/first surgery to death or last follow-up. Multivariate Cox proportional hazards regression analysis was performed in order to evaluate the independent effect of* GPC6* and* TMEM132D* mRNA levels on overall survival. Multivariate regression models included stage, grade, histotype, and* CD8A* mRNA levels. All tests were two-tailed and statistical significance was considered for *p* values < 0.05.

## 3. Results

**Figure 1:**
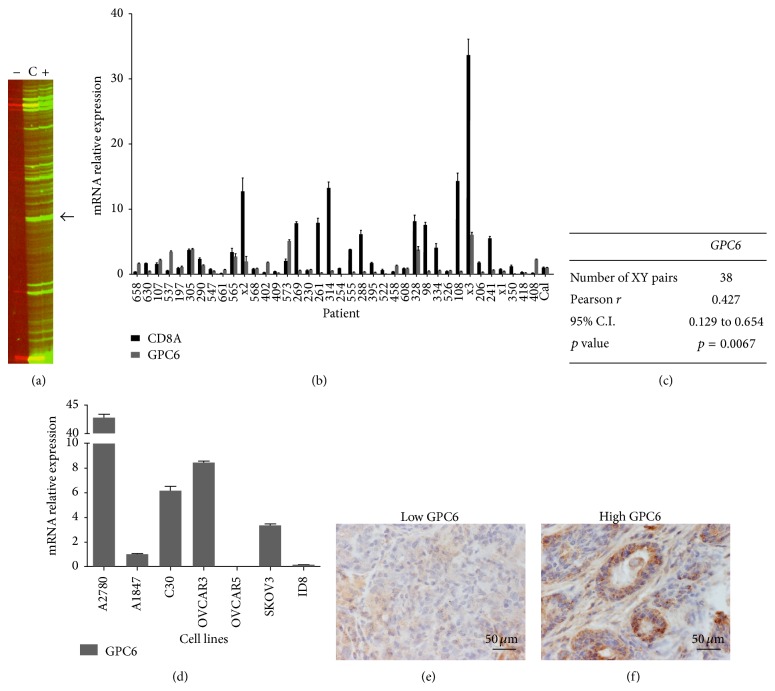
*GPC6* is overexpressed in TIL+ early stage ovarian cancer and its expression correlates positively with CD8+ T-lymphocyte infiltration. (a)* GPC6* was overexpressed in the TIL+ ovarian cancer sample as visualized by fluorescent ADDER (band indicated by an arrow). Lane (+) displays the gene expression profile of the TIL+ sample generated by the corresponding Differential Display primer set; lane (−) displays the expression profile of the TIL− sample and lane (C) displays the combined profile of equal amount of PCR products of both samples. Genes expressed equally in both samples appear as yellow bands in lane (C). (b) The expression of* GPC6* was quantified by qPCR in samples derived from 38 stage I-II ovarian cancer patients. The relative expression of* GPC6* was plotted along with the respective* CD8A* expression levels after normalization to* b-actin*. A pooled mix of equal amounts of all samples served as calibrator (Cal). (c) Pearson correlation coefficient of* CD8A*-*GPC6* expression levels. The correlation is significant (*p* = 0.0067). (d) The relative expression of* GPC6* in 7 ovarian cancer cell lines was estimated by qPCR (normalized to* b-actin*). (e-f) Immunohistochemistry of GPC6 expression in representative early stage ovarian tumor samples. (e) Negative expression of GPC6 protein in the tumor sample 314 with low mRNA levels of the respective gene (see 1(b)). (f) Strong expression of the GPC6 protein in the 408 sample with also high mRNA levels (see 1(b)). Magnification (e-f): ×400.* Columns*: mean,* bars*: SEM.

**Figure 2:**
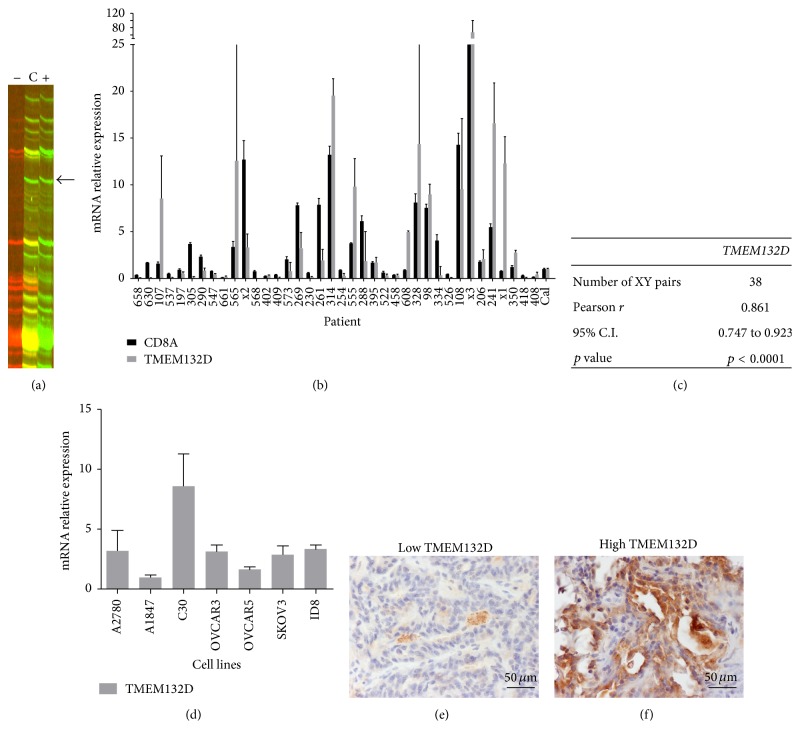
*TMEM132D* is overexpressed in TIL+ early stage ovarian cancer and its expression correlates positively with CD8+ T-lymphocyte infiltration. (a)* TMEM132D* was overexpressed in the TIL+ ovarian cancer sample as visualized by fluorescent ADDER (band indicated by an arrow). (b) The expression of* GPC6* was quantified by qPCR in samples derived from 38 stage I-II ovarian cancer patients. The relative expression of* TMEM132D* was plotted along with the respective* CD8A* expression levels after normalization to* b-actin*. A pooled mix of equal amounts of all samples served as calibrator (Cal). (c) Pearson correlation coefficient of* CD8A*-*TMEM132D* expression levels. The correlation is significant (*p* = 0.0067). (d) The relative expression of* TMEM132D* in 7 ovarian cancer cell lines was estimated by qPCR (normalized to* b-actin*). (e-f) Immunohistochemistry of TMEM132D expression in representative early stage ovarian tumor samples. (e) Negative expression of TMEM132D protein in the tumor sample 408 with low mRNA levels of the respective gene (see 2(b)). (f) Very strong expression of the TMEM132D protein in the 314 sample with also high mRNA levels (see 2(b)). Magnification (e-f): ×400.* Columns*: mean,* bars*: SEM.

By employing a fluorescent version of ADDER Differential Display, we have recently reported the identification of 128 genes overexpressed in early stage ovarian tumors enriched with CD8+ T-lymphocytes (TIL+ tumors) [19].* GPC6* and* TMEM132D*, encoding for the heparan sulfate proteoglycan Glypican-6 and the transmembrane protein 132D, respectively, were included in the identified genes (Figures 1(a) and 2(a), resp.). Here, we further evaluated the expression of these two genes in early stage ovarian cancer.

### 3.1. *GPC6* and* TMEM132D* Are Differentially Expressed in TIL+ Early Stage Ovarian Cancer and Their Expression Levels Correlate with CD8+ T-Cell Infiltration

In order to validate the expression of* GPC6* and* TMEM132D* in early stage ovarian cancer, we measured by qPCR their mRNA levels in tumor samples from 38 stage I-II ovarian cancer patients (Figures 1(b) and 2(b), resp.). The expression of both genes was detectable in all patient samples analyzed, at various levels. To investigate whether* GPC6* and* TMEM132D* expression correlates with CD8+ T-lymphocyte infiltration in early stage ovarian cancer, we used qPCR to quantify the mRNA levels of the* CD8A* marker in the same cohort of patients. Interestingly, a statistically significant positive correlation between the mRNA levels of* GPC6* or* TMEM132D* and* CD8A* accordingly was observed (Figures 1(c) and 2(c), resp.). Thus, the mRNA expression levels of* GPC6* and* TMEM132D* correlate with CD8+ T-lymphocyte infiltration in early stage ovarian cancer. Moreover, we quantified by qPCR the relative expression of* GPC6* and* TMEM132D* in a variety of ovarian cancer cell lines and demonstrated that the expression of both genes is not restricted to the infiltrating immune cells and may actually originate from the tumor cells. As shown in Figure 1(d), expression of* GPC6* was documented in all cell lines tested except one (i.e., OVCAR5). Similarly, all ovarian cancer cell lines expressed* TMEM132D* (Figure 2(c)). To further validate the expression of GPC6 and TMEM132D at protein level in the cancer cells, we performed immunohistochemistry in sections from representative tumor samples with either high or low mRNA levels of* GPC6* and* TMEM132D* as categorized by qPCR (Figures 1(e)-1(f) and 2(e)-2(f)). As depicted in Figure 1(e), the tumors with low mRNA levels of* GPC6* also showed negative expression of the gene at protein level. On the contrary, high expression of the GPC6 protein was observed in tumors with high mRNA levels of the gene. Consistently, the tumors with low mRNA levels of* TMEM132D* showed little expression at protein level, whereas the tumors with high levels of TMEM132D mRNA also showed strong expression at protein level. In conclusion,* GPC6* and* TMEM132D* could serve as potent markers of CD8+ T-cell infiltration in early stage ovarian cancer.

### 3.2. The mRNA Levels of* GPC6* and* TMEM132D* Correlate with Patients' Overall Survival in Early Stage Ovarian Cancer

In order to evaluate the survival predictive value of* GPC6* and* TMEM132D* mRNA levels in early stage ovarian cancer, we performed a retrospective clinical analysis of the above-studied stage I-II ovarian cancer patients. Patients x1, x2, and x3 were excluded from this analysis due to unavailability of complete data. The clinicopathologic characteristics of the 35 early stage ovarian cancer patients are shown in Table 1. Twenty-eight patients (80%) presented stage I ovarian cancer and seven patients (20%) were stage II. The majority of the patients had endometrioid and clear-cell ovarian tumors (Table 1). All of the clear-cell and more than half of the endometrioid tumors (55%) were high grade. Similarly, about 54% of the serous tumours were also high grade. The median age at diagnosis for all patients was 59 years, while the mean overall survival among the entire cohort was 189 months (95% C.I. 164–213, Table 2). Six patients (17.14%) deceased due to the disease during the study period. There were no statistically significant differences in survival among the two stages or the histological subtypes of the disease (data not shown).

As about 25% of the tumors exhibited abundant T-cell infiltration and both* GPC6* and* TMEM132D* correlated with infiltration, the 75th percentile median mRNA levels of* GPC6* (1.8 relative expression units) or* TMEM132D* (5.0 relative expression units) were selected as the cut-off values to subdivide the patient cohort into two groups: patients with high and patients with low mRNA levels of the relevant gene. There was no significant age difference among the two groups of patients, neither in the case of* GPC6* subdivision nor in the case of* TMEM132D* subdivision. The presence of high levels of* GPC6* and* TMEM132D* was also analysed in relation to stage, grade, and type of the disease. Statistically significant differences were not observed for the mRNA levels of the two genes among different stages, grades, or types of tumors (Tables S1 and S2 in Supplementary Material available online at http://dx.doi.org/10.1155/2015/712438). Kaplan-Meir curves were calculated for the mRNA levels in order to assess whether* GPC6* or* TMEM132D* levels were predictive of survival (Figures S1(a) and (b), resp.). As shown in Table 2, the analysis suggested a strong trend towards increased survival for patients with high mRNA levels of* GPC6*. Over twenty percent (21.4%) of the patients with low levels deceased due to the disease. Similar results were obtained for* TMEM132D*. Interestingly, all patients with high mRNA levels of* GPC6* or* TMEM132D* were still alive at the end of the study.

**Figure 3:**
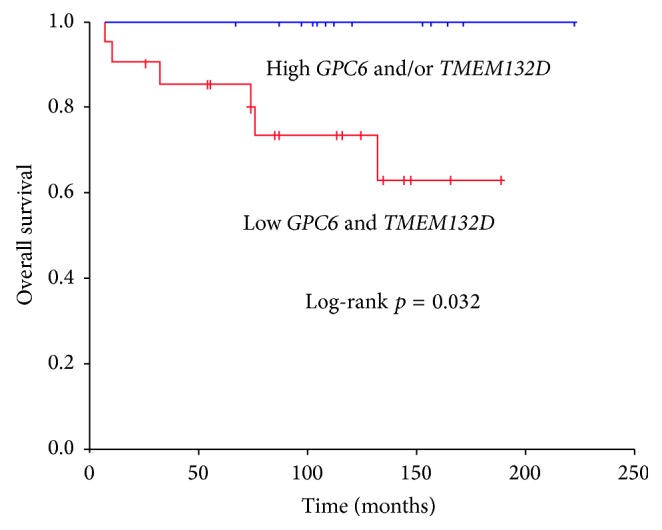
High mRNA levels of* GPC6* and/or* TMEM132D* correlate with increased overall survival of early stage ovarian cancer patients. Kaplan-Meier survival curves calculated for both* GPC6* and* TMEM132D* mRNA levels. Patients were divided into two groups: (i) patients with low mRNA levels of both* GPC6* and* TMEM132D* (cut-off points 1.8 and 5, resp.) and (ii) patients with* GPC6* and/or* TMEM132D* high levels. Patients with high mRNA levels of* GPC6* and/or* TMEM132D* exhibit a favorable overall survival (*p* = 0.032).

We then asked whether the expression of both* GPC6* and* TMEM132D* genes in combination correlated with survival. We divided the patients into 2 groups based on the combined expression of* GPC6* and* TMEM132D*: (i) patients with low levels of both genes (*n* = 22, 62.9%) and (ii) patients with high mRNA levels of* GPC6* and/or* TMEM132D* (*n* = 13, 37.1%). No significant difference in age was observed among the two groups. Patients with low mRNA levels of both* GPC6* and* TMEM132D* had a mean overall survival of 144 months (95% C.I. 114–174). Furthermore, 27.3% of the patients with low mRNA levels deceased due to the disease (Table 2). On the contrary, all the patients with high mRNA levels of* GPC6* and/or* TMEM132D* were alive until the last follow-up (or the end of the study). In conclusion, as shown in Figure 3, high mRNA levels of* GPC6* and/or* TMEM132D* correlate significantly with increased overall survival (*p* = 0.032) in early stage ovarian cancer.

Although CD8A is a classic established marker of T-cell infiltration in ovarian cancer, in our cohort of patients, the prognostic value of* CD8A* mRNA levels was statistically weak (*p* = 0.805) possibly due to the small size of the sample and the low number of deaths. However, in the same cohort of patients, high mRNA levels of* GPC6* and/or* TMEM132* associated significantly with increased survival.

Taken together, our data indicate* GPC6* and* TMEM132D* as potential markers for CD8+ T-lymphocyte infiltration, favorable prognosis, and survival benefit in early stage ovarian cancer.

## 4. Discussion

It is well established that the expression of* CD8A* at the transcriptional level is a reliable indicator of CD8+ T-cell infiltration in ovarian cancer [9, 19]. In this study, we documented an association between the expression of* GPC6* and* TMEM132D* with CD8+ T-lymphocyte infiltration and favorable prognosis in early stage ovarian cancer. Both genes were found to be differentially expressed in TIL+ versus the TIL− early stage ovarian cancer [19]. Here we validated by qPCR analysis in a larger group of stage I/II patients (*n* = 38) that the mRNA expression levels of the two genes significantly correlated with the mRNA levels of the T-cell infiltration marker* CD8A*. More importantly, we showed that early stage ovarian cancer patients with high mRNA levels of at least one of the two genes,* GPC6* and* TMEM132D*, exhibited increased overall survival compared to patients with low levels of both genes.

Our results suggest an involvement of* GPC6* in the biology of lymphocyte infiltration in early stage ovarian tumors.* GPC6* encodes for Glypican-6, a 62.7 kDa heparan sulfate proteoglycan [21, 22]. Glypicans are proteins bearing glycosaminoglycan chains. There are six members of the family (GPC1–GPC6) in mammals, with GPC6 being a close homologue to GPC4 (64% identity). Glypicans are attached to the outer surface of the membrane through a glycosylphosphatidylinositol (GPI) anchor but can also be released to the extracellular space [23, 24]. Their heparan sulfate glycosaminoglycan chains are at the C-terminus of the protein, close to the membrane, and are thought to facilitate the interaction of glypicans with other molecules and receptors of the membrane. It has been shown that glypicans may regulate Hedgehog, Wnt, BMP, and FGF signaling pathways [24]. Interestingly, in* Drosophila* the released glypicans are involved in the transport of Wnts, Hhs, and BMPs by creating a morphogen gradient [25]. Furthermore, GPC6 promotes invasive migration of breast cancer cells through a noncanonical Wnt5A signaling pathway [26]. In particular, NFAT induces the expression of GPC6 which in turn inhibits canonical Wnt and b-catenin signaling and activates Wnt5A signaling that results in activation of JNK and p38*α* kinases [26]. Further studies are required in order to shed light on the role of GPC6 in ovarian cancer and the mechanism of CD8+ T-lymphocyte infiltration. It is reasonable to anticipate that GPC6 may mediate intercellular interactions with immune cells or that it may be involved in shaping a chemokine gradient, necessary for the CD8+ T-lymphocyte infiltration.

On the other hand, little is known about the biological role of* TMEM132D*. Polymorphisms in* TMEM132D* gene have been associated with panic disorder [27, 28].* TMEM132D* encodes a single-pass type I transmembrane protein initially discovered in mature oligodendrocytes.* TMEM132D* expression is detectable in brain, lung, pancreas, and testis, but intriguingly not in normal ovaries [29]. Thus, it is possible that the expression of* TMEM132D* is induced in ovarian cancer and by an unknown mechanism becomes implicated in CD8+ T-lymphocyte infiltration. Based on the presence of TMEM132D on the plasma membrane surface, one could hypothesize an involvement in cell-cell interactions or intercellular signaling mechanisms that could be implicated with T-cell recruitment.

According to our results, early stage ovarian cancer patients with low mRNA levels of* GPC6* and* TMEM132D* exhibit significantly reduced overall survival compared to patients with high levels of* GPC6* and/or* TMEM132D*. Therefore, we suggest that* GPC6* and* TMEM132D* mRNA levels could serve as markers of CD8+ T-cell infiltration and survival prognosis in early stage ovarian cancer. For example, monitoring tumoral* GPC6* and* TMEM132D* mRNA levels could facilitate the identification of early stage ovarian cancer patients at high risk. Our conclusions underscore the necessity to elucidate the molecular mechanism of* GPC6* and* TMEM132D* involvement in T-cell infiltration and their impact on cancer progression and also highlight their possible importance as putative diagnostic/therapeutic targets.

## Supplementary Material

Supplementary Figure S1: Kaplan-Meier curve analysis suggest a trend towards higher overall survival for patients with high mRNA levels of GPC6 or *TMEM132D*. Kaplan-Meier overall survival curves calculated for GPC6 (A) and *TMEM132D* (B) tumoral mRNA levels in 35 early stage ovarian cancer patients. The patients were divided into two groups based on their GPC6 mRNA levels (A, cut-off point: 1.8 relative expression units) or their *TMEM132D* mRNA levels (B, cut-off point: 5.0 relative expression units).
Supplemental Table S1: Association of GPC6 mRNA level with the stage, grade and histotype of the 35 early stage tumors.
Supplemental Table S2: Association of *TMEM132D* mRNA level with the stage, grade and histotype of the 35 early stage tumors.

## Figures and Tables

**Table 1:** Clinicopathologic characteristics of the stage I/II ovarian cancer patients.

Characteristic	*N* (%)		
	All stages, *N* = 35	Stage I, *N* = 28 (80%)	Stage II, *N* = 7 (20%)
*Age at diagnosis *			
Median/mean	59/57.63	59/56.86	59/60.71
Range	(34–83)	(34–78)	(51–83)
*Grade *			
0	2 (5.7)	2 (7.1)	
1	12 (34.3)	10 (35.7)	2 (28.6)
2	8 (22.9)	5 (17.9)	3 (42.9)
3	13 (37.1)	11 (39.3)	2 (28.6)
*Histological subtype *			
Serous	11 (31.4)	9 (32.1)	2 (28.5)
Endometrioid	20 (57.2)	16 (57.2)	4 (57.2)
Clear-cell	4 (11.4)	3 (10.7)	1 (14.3)
*Debulking status *			
Optimal	34 (97.1)	28 (100)	6 (85.7)
Suboptimal	1 (2.9)		1 (14.3)
*Response to therapy *			
CR^a^	33 (94.3)	27 (96.4)	6 (85.7)
PD^b^	2 (5.7)	1 (3.6)	1 (14.3)
*Survival *			
Ovarian cancer deaths	6 (17.1)	4 (14.3)	2 (28.5)
Total number of deaths	6 (17.1)	4 (14.3)	2 (28.5)

^a^Complete response.

^b^Progressive disease.

**Table 2:** Survival characteristics of patients related to their tumoral *GPC6* and *TMEM132D* mRNA levels.

	All	GPC6 mRNA levels		TMEM132D mRNA levels		GPC6/TMEM132D mRNA levels	
		Low	High	Low	High	Both low	High GPC6 and/or TMEM132D
*N (%) *	35	28 (80)	7 (20)	26 (74.3)	9 (25.7)	22 (62.9)	13 (37.1)
*Survival *							
5-year survival (%)	91.34 ± 4.78	88.73 ± 6.13	100	88.29 ± 6.36	100	85.45 ± 7.78	100
10-year survival (%)	84.69 ± 6.34	80.06 ± 8.04	100	78.73 ± 8.55	100	73.62 ± 10.27	100
*Overall survival (months) *							
Mean ± SE	189 ± 12	179 ± 15	Undefined^a^	152 ± 13	Undefined^a^	144 ± 15	Undefined^a^
95% C.I.	164–213	149–209	—	126–177	—	114–174	—
*Death cases* (%)	6 (17.14%)	6 (21.4)	0 (0)	6 (23.1)	0 (0)	6 (27.3)	0 (0)

*p* value (log-rank test)		0.214		0.099		0.032	

^a^All subjects alive.
