# Supplementary material for: Overexpression of GPC6 and TMEM132D in Early Stage Ovarian Cancer Correlates with CD8+ T-Lymphocyte Infiltration and Increased Patient Survival
Source: Biomed Res Int. 2015 Sep 13;2015:712438. doi: 10.1155/2015/712438 (PMC4584051; doi:10.1155/2015/712438)
Supplement: Supplementary file 1 — Supplementary Figure S1: Kaplan-Meier curve analysis suggest a trend towards higher overall survival for patients with high mRNA levels of GPC6 or TMEM132D. Kaplan-Meier overall survival curves calculated for GPC6 (A) and TMEM132D (B) tumoral mRNA levels in 35 early stage ovarian cancer patients. The patients were divided into two groups based on their GPC6 mRNA levels (A, cut-off point: 1.8 relative expression units) or their TMEM132D mRNA levels (B, cut-off point: 5.0 relative expression units). Supplemental Table S1: Association of GPC6 mRNA level with the stage, grade and histotype of the 35 early stage tumors. Supplemental Table S2: Association of TMEM132D mRNA level with the stage, grade and histotype of the 35 early stage tumors. [file 712438.f1.pdf]

Figure S1

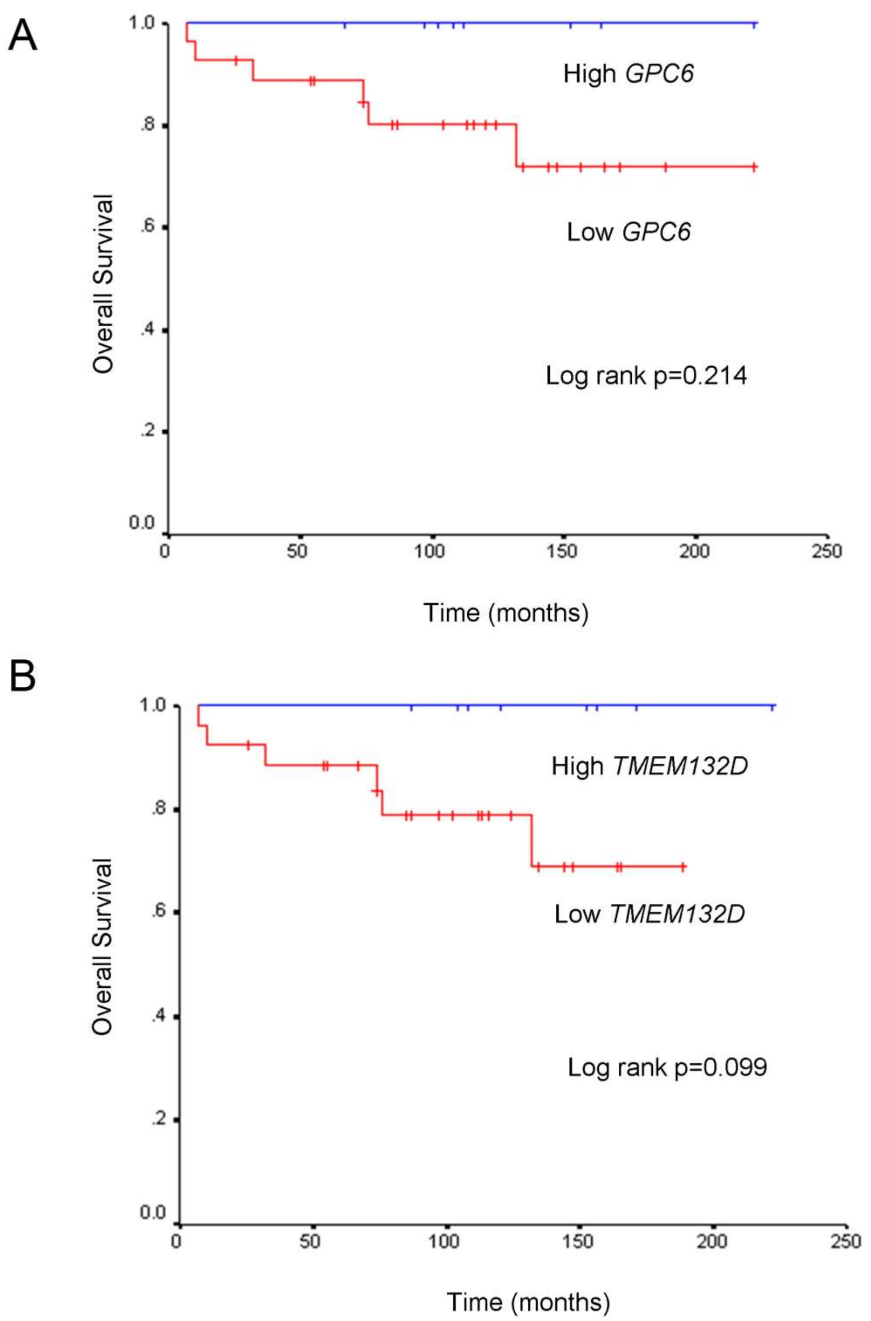
A
1.0
.8
.6
.4
.2
0.0
Overall Survival
0
50
100
150
200
250
Time (months)
High *GPC6*
Low *GPC6*
Log rank p=0.214
B
1.0
.8
.6
.4
.2
0.0
Overall Survival
0
50
100
150
200
250
Time (months)
High *TMEM132D*
Low *TMEM132D*
Log rank p=0.099

Table S1: Association of *GPC6* mRNA level with the stage, grade and histotype of the 35 early stage tumors.

	Stage		Grade			Histological subtype		
	**I**	**II**	**0-1**	**2**	**3**	**Endometrioid**	**Serous**	**Clear cell**
***GPC6* high (*N*=7)**	6 (21.4%)	1 (14.3%)	5 (35.7%)	1 (12.5%)	1 (7.7%)	6 (30%)	1 (9.1%)	0 (0%)
***GPC6* low (*N*=28)**<br>Total *N*=35	22 (78.6%)	6 (85.7%)	9 (64.3%)	7 (87.5%)	12 (92.3%)	14 (70%)	10 (90.0%)	4 (100%)
p-value (chi-square)	0.673		0.159			0.216		

The relationship of high or low mRNA levels of *GPC6* with clinicopathologic characteristics (stage, grade, histotype) were analyzed by Chi-squared test. Statistically significant associations were not observed.

Table S2: Association of *TMEM132D* mRNA level with the stage, grade and histotype of the 35 early stage tumors.

	Stage		Grade			Histological subtype		
	**I**	**II**	**0-1**	**2**	**3**	**Endometrioid**	**Serous**	**Clear cell**
***TMEM132D* high (*N*=9)**	7 (25%)	2 (28.6%)	3 (21.4%)	4 (50%)	2 (15.4%)	6 (30%)	2 (18.2%)	1 (25%)
***TMEM132D* low (*N*=26)**<br>Total *N*=35	21 (75%)	5 (71.4%)	11 (78.6%)	4 (50%)	11 (84.6%)	14 (70%)	9 (81.8%)	3 (75%)
p-value (chi-square)	0.847		0.189			0.771		

The relationship of high or low mRNA levels of *TMEM132D* with clinicopathologic characteristics (stage, grade, histotype) were analyzed by Chi-squared test. Statistically significant associations were not observed.
